# Physician-related determinants of medical end-of-life decisions – A mortality follow-back study in Switzerland

**DOI:** 10.1371/journal.pone.0203960

**Published:** 2018-09-20

**Authors:** Matthias Bopp, Yolanda W. H. Penders, Samia A. Hurst, Georg Bosshard, Milo A. Puhan, for the Swiss End-of-Life Decisions Study Group

**Affiliations:** 1 Epidemiology, Biostatistics and Prevention Institute, University of Zurich, Zürich, Switzerland; 2 Institute for Ethics, History, and the Humanities, Geneva University Medical School, Genève, Switzerland; 3 Clinic for Geriatric Medicine, Zurich University Hospital, and Center on Aging and Mobility, University of Zurich and City Hospital Waid, Zürich, Switzerland; Foundation IRCCS Neurological Institute C. Besta, ITALY

## Abstract

**Background:**

Medical end-of-life decisions (MELD) and shared decision-making are increasingly important issues for a majority of persons at the end of life. Little is known, however, about the impact of physician characteristics on these practices. We aimed at investigating whether MELDs depend on physician characteristics when controlling for patient characteristics and place of death.

**Methods and findings:**

Using a random sample (N = 8,963) of all deaths aged 1 year or older registered in Switzerland between 7 August 2013 and 5 February 2014, questionnaires covering MELD details and physicians' demographics, life stance and medical formation were sent to certifying physicians. The response rate was 59.4% (N = 5,328). Determinants of MELDs were analyzed in binary and multinomial logistic regression models. MELDs discussed with the patient or relatives were a secondary outcome. A total of 3,391 non-sudden nor completely unexpected deaths were used, 83% of which were preceded by forgoing treatment(s) and/or intensified alleviation of pain/symptoms intending or taking into account shortening of life. International medical graduates reported forgoing treatment less often, either alone (RRR = 0.30; 95% CI: 0.21–0.41) or combined with the intensified alleviation of pain and symptoms (RRR = 0.44; 0.34–0.55). The latter was also more prevalent among physicians who graduated in 2000 or later (RRR = 1.60; 1.17–2.19). MELDs were generally less frequent among physicians with a religious affiliation. Shared-decision making was analyzed among 2,542 decedents. MELDs were discussed with patient or relatives less frequently when physicians graduated abroad (OR = 0.65, 95% CI: 0.50–0.87) and more frequently when physicians graduated more recently; physician's sex and religion had no impact.

**Conclusions:**

Physicians' characteristics, including the country of medical education and time since graduation had a significant effect on the likelihood of an MELD and of shared decision-making. These findings call for additional efforts in physicians' education and training concerning end-of-life practices and improved communication skills.

## Introduction

Compared to other countries, an exceptionally high percentage of deaths in Switzerland is preceded by a decision to forgo life-prolonging treatments [1–3], contributing to a high overall prevalence of medical end-of-life decisions (MELDs). These differences between Switzerland and other countries may be explained by what Gysels et al. called "evidence for clearly distinguishable national cultures of end-of-life care, with differences in meaning, priorities, and expertise in each country" [4]. In addition to such cultural factors, previous research has also shown that certain groups of patients are more likely to experience death preceded by an MELD, particularly people who die of cancer and older people [5,6] and that there are differences in the type of MELD by patient’s sex and age [6–8]. Likewise, the involvement of patients or relatives in the decision-making process has been shown to be influenced by cultural and patient-related factors, such as age [8–10].

There are several ways in which also a physician’s characteristics could affect medical end-of-life decision-making and the discussion of these decisions with patients or relatives. However, a 2011 systematic review of the literature on patient and healthcare professional factors influencing end-of-life decision-making concluded that only a few studies examined the influence of physician-specific factors and those that did often focused on decision-making in hospital or acute care units only [11]. In Belgium MELDs were more frequent among physicians having received a postgraduate training in palliative or terminal care and among those having attended a non-Catholic university [6]. Younger physicians have also been reported to involve patients more often in discussions around MELDs [6,9]. Most studies, however, only assessed attitudes or evaluated hypothetical patients. In a U.S. study, Catholic physicians had significantly more objections to the withdrawal of life support than their Protestant peers [12] and in an international study, physicians with specific religious affiliations showed less willingness than non-religious physicians to administer drugs explicitly intending to hasten patient's death [13]. There is evidence that physician’s sex has an important impact on the time spent on communication [14] and on health outcomes [15]. Female physicians were reported to be less supportive than male physicians towards ending of life without explicit request, more supportive of intensified alleviation of pain and symptoms with possible life-shortening effect [16]. There is evidence that female physicians are more likely to engage in patient-centered communication [14], an important precondition for shared decision-making. Quite unsurprisingly, health outcomes also vary by physician's years of practice [17,18].

Cultural or country-specific factors could also influence the training and education of physicians. Depending on where a physician was trained, there may be not only variation regarding outcomes [17], but also whether a physician is more or less likely to make an MELD. Such cultural influence may also affect the likelihood of a physician involving patients and relatives in a discussion about MELDs. A study on end-of-life decision-making in an Israeli intensive care unit found that whether physicians had trained in America or in Eastern Europe had a large impact on how often they discussed forgoing life-sustaining treatments with patients [19]. Further evidence for the potential impact of physician’s characteristics on shared decision-making came from a systematic review that found that one of the biggest facilitators for shared-decision making was the motivation of health professionals and their view that shared-decision making would lead to better patient outcomes [20].

The lack of knowledge surrounding how physician’s characteristics impact real decision-making at the end of life–both the types of decision made as the involvement of others in this decision–makes it hard to address potential inequalities in decision-making, and to ensure shared decision-making is a priority in all end-of-life situations. With this study we aim to assess the impact of physician-related determinants of MELDs and patient involvement in these decisions. There are two specific research questions. First: Do physician characteristics have an impact on the likelihood of an MELD when controlling for both patient and setting characteristics? Second, do physician characteristics–in case an MELD took place–have an impact on the likelihood of patient and/or relatives being involved in shared decision-making when controlling for both the patient and setting characteristics?

## Methods

### Data collection

We conducted a mortality follow-back study on a continuous random sample of death registrations in Switzerland between August 7, 2013 and February 5, 2014, from which we obtained from the Swiss Federal Statistical Office the address of the certifying physician. The sample represented 21.3% of deaths among those aged 1 year or older in the German-, 41.1% in the French- and 62.9% in the Italian-speaking regions of Switzerland [21]. In total, 8,963 questionnaires were mailed in a strictly anonymous setting to the certifying physicians, of which 5,328 (59.4%) were returned until June 11, 2014.

### Measures

Using an internationally standardized questionnaire [5] (online English version: [22]), physicians were asked whether they had: (1) withheld or withdrawn a probably life-prolonging medical treatment taking into account the possibility of hastening the patient’s death or explicitly intending to hasten the patient's death or not to prolong their life; (2) intensified the alleviation of pain and/or symptoms (APS) with drugs taking into account or partly intending hastening the patient's death; or (3) prescribed or administered a drug with the explicit intention of ending the patient's life. For all these cases it was also assessed whether the patient ever expressed a wish for hastening death or for applying all life-prolonging measures, and whether the MELD was discussed with the patient or other persons (relatives, other physicians, healthcare professionals, any other person). Continuous deep sedation was assessed separately, but no questions were asked about the decision-making process so it is not included in this paper.

The region was defined by the language of the death certificate and place of death was determined from the categories in the questionnaire (at home, retirement community, long-term care home, hospice or palliative unit, hospital, other place).

Patient characteristics were available from death certificates (sex, age, civil status, religious affiliation). Broad cause of death was assessed in the questionnaire, because the cause of death information was not yet available from this early version of the death certificates and–due to anonymization–could not be supplemented ex post. More details are given elsewhere [23].

The questionnaire also encompassed several questions about the attending physician, namely sex, year (before 1970, 1970–1984, 1985–1999, 2000 or later) and place of graduation (German-speaking Switzerland, French-speaking Switzerland, abroad), the number of deceased patients cared for in the preceding six months, palliative care education (yes/no), religion or life stance (catholic, protestant, other religion/philosophy, not religious) and importance of religion/life stance for making an MELD (very important, important, less important, not important).

### Sample

We selected all patients who were permanent residents of Switzerland, did not die suddenly and completely unexpected or by assisted suicide, and had a first contact with the responding physician when still alive. Out of 5,328 returned questionnaires, 3,391 concerned deaths that fulfilled these criteria and had at least minimal information on place of death and important physician attributes (sex, place and year of graduation).

For the analysis of shared decision-making, deaths without preceding forgone treatment or APS (N = 619) or lacking all information regarding discussion as well as other expression of patient's preferences (N = 230) were excluded, leaving 2,542 decedents with forgone treatment and/or APS and information about shared-decision making.

### Statistical analysis

An age-sex-region-specific weighting was applied to make the data representative of all deaths in the sample period. Weighted percentages were used to describe the data.

Multinomial logistic regression [24] was used to calculate relative risk ratios (RRR), 95% confidence intervals (CI) and P values of potential determinants of different MELD categories. Binary logistic regression was used to calculate odds ratios, 95% confidence intervals (CI) and P values of potential determinants of any MELD vs. no MELD as well as of patient and family involvement in MELDs. All calculations were performed using Stata (version 13.1, StataCorp) statistical package.

### Ethics approval and consent to participate

The study was declared exempt from ethics review by the Zurich Cantonal Ethics Board (KEK-StV-Nr. 23/13). Participants were informed about the study in a cover letter. Questionnaire return was considered to imply consent to participate.

## Results

### Medical end-of-life decisions

Out of 3,391 eligible deaths, 2,772 (83%) were preceded by forgoing a life-prolonging treatment (17% of cases), intensified alleviation of pain and symptoms (APS; 12%) or both measures combined (54%; Table 1).

**Table 1:** Descriptives of the study population: Forgoing treatment(s)* and APS** in Switzerland 2013–2014 (N = 3,391).

	Forgoing* alone		Forgoing* & APS**		APS** alone		Neither forgoing nor APS	
	N = 535 (16.6%)		N = 1801 (53.9%)		N = 436 (12.3%)		N = 619 (17.2%)	
	N	%	N	%	N	%	N	%
**Patient’s characteristics**								
Patient’s sex: Female	286	16.7	961	53.8	253	13.3	308	16.2
Male	249	16.5	840	53.9	183	11.1	311	18.4
Patient’s age: <65y	59	15.4	222	56.7	45	11.2	72	16.7
65-79y	130	15.3	469	54.4	102	11.6	166	18.7
80y and over	346	17.3	1110	53.2	289	12.8	381	16.7
Patient's nationality: Swiss	494	16.8	1644	53.7	399	12.4	558	17.1
Foreign	41	14.3	157	55.9	37	11.5	61	18.3
Patient's religion: Non-religious	34	19.0	96	54.6	20	9.8	31	16.6
Catholic	218	16.5	729	53.8	184	12.6	246	17.1
Protestant	210	16.6	701	54.9	161	12.3	220	16.2
Other^†^	73	15.8	275	50.5	71	12.6	122	21.1
Patient's civil status: Married	212	16.9	720	54.6	171	12.0	237	16.5
Single	50	14.7	185	55.3	38	12.2	67	18.0
Widowed	229	17.5	720	53.4	191	12.9	232	16.2
Divorced	44	13.4	176	51.9	36	11.1	83	23.6
Cause of death: CVD	152	18.9	389	47.6	107	13.0	182	20.4
Injury/unknown	31	25.8	59	44.6	14	9.3	29	20.3
Cancer	104	10.7	617	58.0	158	13.5	203	17.8
Other	248	18.6	736	55.7	157	11.2	205	14.5
**Physician’s characteristics**								
Physician’s sex: Female	168	15.2	637	57.7	142	12.3	181	14.8
Male	367	17.3	1164	52.0	294	12.3	438	18.4
Year of graduation: <1985	204	19.5	559	48.8	168	13.6	222	18.1
1985–1999	134	17.1	425	51.8	100	12.2	162	18.9
≥2000	197	14.0	817	59.2	168	11.3	235	15.5
Place of graduation: Switzerland	463	18.4	1442	54.9	315	15.9	431	15.3
Outside of Switzerland	72	9.8	359	50.1	121	11.3	188	24.2
Physician's religion: Non-religious	155	15.9	594	58.4	124	11.8	150	14.0
Catholic	150	14.7	565	52.3	157	13.6	227	19.2
Protestant	153	18.4	464	54.9	89	10.2	146	16.5
Other^†^	77	19.1	178	43.8	66	15.3	96	21.8
**Setting characteristics**								
Place of death: Hospital^‡^	229	15.0	870	57.1	180	11.4	272	16.6
Home setting/other^§^	132	18.5	382	47.6	119	13.7	174	20.1
Long-term care home	174	17.5	549	53.9	137	12.6	173	16.0

*: Withholding or withdrawing treatment

**: Intensified alleviation of pain and symptoms

†: Includes ‘no answer’

‡: Includes hospital and palliative care unit/hospice

§: Includes home, elderly care residence, and unspecified ‘other’

The estimated relative risk ratios (RRR) from a multinomial logistic regression model contrasting "forgoing alone", "APS alone" and "both measures combined" with "neither" as reference category are presented in Table 2, along with 95% confidence intervals. Patient's nationality and religion were dropped in the final model, because they did not contribute information.

**Table 2:** Patient and physician related determinants for medical end-of-life decisions: multinomial logistic regression with neither forgoing nor APS (N = 619) as reference category adjusted for cause of death, place of death, language region and patients' age.

	Forgoing* alone		Forgoing* & APS˚		APS˚ alone	
	N = 535		N = 1801		N = 436	
	RRR (95% CI)	P-value	RRR (95% CI)	P-value	RRR (95% CI)	P-value
**Patient’s characteristics**						
Sex: Female	*Ref*		*Ref*		*Ref*	
Male	0.89 (0.69–1.15)	0.38	0.87 (0.70–1.07)	0.18	0.74 (0.56–0.97)	0.03
Civil status: Married	*Ref*		*Ref*		*Ref*	
Single	0.72 (0.47–1.11)	0.14	0.89 (0.64–1.25)	0.51	0.82 (0.52–1.30)	0.40
Widowed	0.94 (0.70–1.26)	0.68	1.02 (0.80–1.29)	0.90	0.98 (0.71–1.34)	0.89
Divorced	0.56 (0.37–0.86)	<0.01	0.65 (0.47–0.89)	<0.01	0.59 (0.38–0.92)	0.02
**Physician’s characteristics**						
Year of graduation <1985	*Ref*		*Ref*		*Ref*	
1985–1999	0.90 (0.66–1.23)	0.50	1.04 (0.81–1.34)	0.76	0.85 (0.60–1.18)	0.33
≥2000	0.98 (0.67–1.44)	0.91	1.60 (1.17–2.19)	<0.01	0.98 (0.65–1.48)	0.94
Graduation: in Switzerland	*Ref*		*Ref*		*Ref*	
Outside of Switzerland	0.30 (0.21–0.41)	<0.001	0.44 (0.34–0.55)	<0.001	0.86 (0.62–1.17)	0.33
Sex: Female	*Ref*		*Ref*		*Ref*	
Male	0.80 (0.60–1.06)	0.12	0.80 (0.64–1.01)	0.06	0.78 (0.57–1.05)	0.10
Religion: Non-religious	*Ref*		*Ref*		*Ref*	
Catholic	0.71 (0.52–0.98)	0.04	0.72 (0.56–0.92)	<0.01	0.85 (0.61–1.18)	0.33
Protestant	0.88 (0.64–1.22)	0.45	0.78 (0.60–1.01)	0.06	0.70 (0.49–1.00)	0.05
Other†	0.86 (0.59–1.27)	0.45	0.51 (0.37–0.71)	<0.001	0.85 (0.56–1.27)	0.42

*Withholding or withdrawing treatment.

˚Intensified alleviation of pain and symptoms.

†Includes ‘no answer’.

Compared to physicians that graduated from a Swiss university, those who graduated from abroad reported substantially less often forgoing treatment alone (RRR: 0.30; 95% CI: 0.21–0.41) or combining forgoing treatment with APS (0.44; 0.34–0.55) while for APS alone there was only a marginal effect. Having graduated in 2000 or later had only an effect for the combination of forgoing and APS (1.60; 1.17–2.19), but not for the two "alone" categories. Forgoing life-prolonging treatment alone or combined with APS was less prevalent when physicians reported Catholic as their religious affiliation (0.71 / 0.72; 0.52–0.98 / 0.56–0.92), whereas among Protestant physicians the lower prevalence was predominantly driven by APS. The lowest prevalence however was found for the combination of forgoing and APS among physicians reporting another religious affiliation or philosophy of life (0.51; 0.37–0.71). Male physicians tended to report MELDs less frequently than their female colleagues. In all MELD categories, divorced patients substantially less often experienced an MELD (RRRs between 0.56 and 0.65).

**Fig 1:**
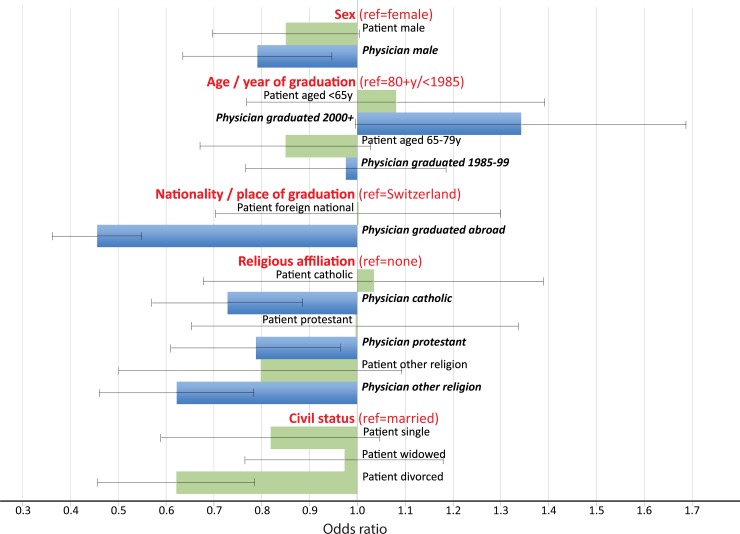
Patient- vs. physician-related determinants for medical end-of-life decisions (forgoing treatment(s) and/or intensified alleviation of pain): Multiple logistic regression (N = 3,391). Additionally adjusted for cause of death category, place of death and language region. Patient-related variables are mapped in bright green and physician-related variables in dark blue. Bars indicate 95% confidence intervals.

It is tempting to line-up the impact of corresponding physician and patient determinants like sex, age, religious affiliation and foreign origin. The results of a multiple binary logistic regression model (adjusted additionally for the cause of death, place of death and language region) show that physician-related determinants had generally a larger effect size than patient-related ones (Fig 1). Compared to physicians that graduated from a Swiss university, those who graduated abroad reported significantly less often forgoing treatment or APS, whereas nationality of the patient was irrelevant. This contrast between a substantial effect among physicians but only minor effects among decedents also applied to religious affiliation. Only sex showed some similarity: Male patients and even more so patients cared for by a male physician were less likely to die following forgoing treatment or APS.

### Patient and family involvement in medical end-of-life decisions

Most MELDs (79%) were discussed with the patient directly or with their relatives (Table 3).

**Table 3:** Descriptives of the study population: Discussion of forgoing treatment and/or APS with patient and/or relatives (N = 2,542).

	Discussed with patient		Discussed with relatives, not with patient		Not discussed with patient or relatives	
	N	%	N	%	N	%
**Total**	916	37.3	1087	41.4	539	21.2
**MELD category**						
Forgoing* alone	181	37.3	213	41.2	108	21.5
Forgoing* and APS˚ combined	689	41.1	779	43.4	269	15.5
APS˚ alone	46	14.9	95	29.7	162	55.4
**Patient’s characteristics**						
Patient’s sex: Female	478	36.2	580	40.7	310	23.2
Male	438	38.8	507	42.3	229	18.9
Patient’s age: <65y	125	42.7	113	35.8	68	21.5
65-79y	266	42.5	265	40.1	114	17.4
80y and over	525	34.4	709	42.9	357	22.6
Patient's nationality: Swiss	837	37.2	989	41.1	502	21.7
Foreign	79	38.6	98	45.5	37	15.9
Patient's religion: Non-religious	61	44.9	48	34.1	30	21.0
Catholic	370	37.1	467	43.8	197	19.1
Protestant	368	37.4	398	39.9	222	22.7
Other†	117	34.2	174	42.7	90	23.1
Patient's civil status: Married	418	42.3	434	42.0	165	15.7
Single	77	30.3	90	33.7	87	36.0
Widowed	344	34.7	476	44.1	221	21.2
Divorced	77	35.0	87	35.1	66	29.9
Cause of death						
Cardiovascular diseases	198	34.9	257	42.0	133	23.0
Injury/unknown	16	18.3	57	60.7	19	21.0
Cancer	405	53.4	230	26.3	169	20.3
Other	297	29.1	543	50.0	218	20.9
**Physician’s characteristics**						
Physician’s sex: Female	339	39.6	367	42.0	161	18.4
Male	577	36.1	720	41.1	378	22.8
Year of graduation: <1985	239	29.6	374	42.2	237	28.6
1985–1999	223	38.6	251	40.2	125	21.2
>2000	454	42.6	462	41.5	177	15.9
Place of graduation: Switzerland	730	36.7	897	42.5	423	20.8
Outside Switzerland	186	40.2	190	36.7	116	23.1
Physician’s religion: Non-religious	302	38.0	351	42.9	153	19.1
Catholic	253	34.4	366	43.7	174	21.9
Protestant	255	39.8	248	37.0	149	23.1
Other†	106	37.0	122	42.1	63	20.9
**Place of death**						
Hospital‡	495	42.8	507	41.3	194	15.9
Long-term care home	231	30.3	351	43.6	201	26.1
Home setting/other§	190	36.2	229	38.3	144	25.5

Notes

*Withholding or withdrawing treatment.

˚Intensified alleviation of pain and symptoms.

†Includes ‘no answer’.

‡Includes hospital and palliative care unit/hospice.

§Includes home, elderly care residence, and unspecified ‘other’.

Involvement of the patient was more frequent among cancer patients than on average (53% vs. 37%). In multiple binary logistic regression analysis (not differentiating whether an MELD was discussed directly with the patient or only with relatives), however, there was no substantial variation between the broad cause of death groups. Nevertheless, the type of MELD had a large influence: Compared to forgoing a life-prolonging treatment alone, shared decision-making was substantially more frequent for combined forgoing and APS (OR: 1.51; 95% CI: 1.18–1.95) and much less frequent for APS alone (0.21; 0.15–0.29)(Table 4).

**Table 4:** Involvement of patient and/or relative(s) in medical end-of-life decisions (MELD): Multiple logistic regression (N = 2,542).

	MELD discussed with patient and/or relative(s)	
	OR (95% CI)	P-value
**MELD category**		
Forgoing* alone	*Ref*	
Forgoing* and APS˚ combined	1.51 (1.18–1.95)	<0.01
APS˚ alone	0.21 (0.15–0.29)	<0.001
**Patient’s characteristics**		
Civil status: Married	*Ref*	
Single	0.32 (0.23–0.45)	<0.001
Widowed	0.79 (0.62–1.00)	0.05
Divorced	0.41 (0.29–0.58)	<0.001
**Physician’s characteristics**		
Year of graduation: <1985	*Ref*	
1985–1999	1.34 (1.03–1.75)	0.03
>2000	1.73 (1.27–2.37)	<0.01
Place of graduation: Switzerland	*Ref*	
Outside Switzerland	0.65 (0.50–0.87)	<0.01
**Place of death**		
Hospital^‡^	*Ref*	
Long-term care home	0.69 (0.50–0.96)	0.03
Home setting/other^§^	0.62 (0.46–0.83)	<0.01

*Withholding or withdrawing treatment.

˚Intensified alleviation of pain and symptoms.

^‡^Includes hospital and palliative care unit/hospice.

^§^Includes home, elderly care residence, and unspecified ‘other’.

Discussing decisions with the patient or relatives was significantly more likely when physicians graduated more recently (in 1985–1999 / in 2000 or later: 1.34; 1.03–1.75 / 1.73; 1.27–2.37). Conversely, shared decision-making was significantly less likely when physicians graduated outside of Switzerland (0.65; 0.50–0.87) or when patients died elsewhere than in a hospital. Physician's sex and religion or philosophy had no influence on the frequency of discussing decisions with the patient or relatives and were dropped in the final model. Compared to married decedents, shared decision-making was less frequent when patients were single (0.32; 0.23–0.45) or divorced (0.41; 0.29–0.58), but not when they were widowed. Patient's sex, age, nationality and religious affiliation were irrelevant and were also dropped in the final model.

The other potential physician determinants (palliative care education, the importance of religion/life stance for making an MELD, number of deceased patients cared for) were tested but did not have a significant impact on either the likelihood of forgoing and/or APS or the involvement of patients and relatives in decision-making.

## Discussion

Even when adjusting for patient and setting characteristics, physician's characteristics had a substantial impact on MELD practices and the prevalence of shared decision-making in our study population, suggesting a sizable potential for optimizing end-of-life care.

International medical graduates made substantially fewer MELDs than those who graduated from a Swiss university and when they made an MELD, they less often reported shared decision-making with the patient and/or their relatives. International medical graduates may be less familiar with shared decision-making and patient-centered care [25]. At the time of the survey, 29% of all physicians in Switzerland were international graduates, with almost 60% of them originating from Germany [26, 27]. A study revealed that German physicians chose more life-prolonging interventions than their Swedish peers [28] and a scoping review stated that "German physicians were found to be more likely to exclude patients, patients' families and non-medical staff from the decision-making process" [4]. In another study, it was shown that where physicians were trained had a significant impact on how often they discussed forgoing treatment with patients [19]. These findings support the notion that physicians’ medical education may have a long-lasting impact on their attitudes towards care and decision-making. Physicians who graduated more recently made more MELDs and discussed these more often with patients and relatives than their colleagues who graduated before 1985. Similarly, a Belgian study found in the late 1990s that MELDs were significantly less frequent among patients treated by GPs aged 55 years and older [29]. This may also be due to previously lacking education in ethics: In Switzerland the first compulsory ethics classes started in 1995 only [30]. Ethics education has been described as supporting physicians' confidence regarding procedural end-of-life issues [31] and as being associated with a higher likelihood of applying a written do not resuscitate order [32]. Physicians inclined to apply "full code" had less often read about end-of-life care and had less interest in discussing MELDs than physicians more disposed to withdrawing life-sustaining therapies [32]. However, a 2002 study showed that ethics education was not associated with confidence in decisions to withdraw life support after an intensive care unit rotation and argued instead for experiential, case-based, patient-centred curricula for physicians-in-training [33].

Moreover and independently of age and place of graduation, physicians' religion mattered: physicians with a religious affiliation made fewer MELDs than those without. This is in line with a former study in Switzerland exploring attitudes regarding hypothetical patients and where religious believers tended to disagree more often with end-of-life decisions than other doctors [34]. On an international level, evidence for an impact of religious belief on the general incidence of MELDs is rather weak and controversial [12]. There is more evidence, however, for non-religious physicians being more inclined to make MELDs that may result in the hastening of death [16,29,35,36], and for a larger impact of religion for more drastic life-shortening acts [13]. Of note, the importance attached to religion when making an MELD had no influence on real patterns in our study and did not support the expectations of intrinsic religiosity of physicians playing a major role [12,34]. In contrast to our expectations, physician's sex had an only marginal influence on MELD incidence and no impact at all regarding the prevalence of shared decision-making. Nevertheless, future research should not discount the possibility of an effect of gender.

Except for the cause of death, civil status was the only patient characteristic with an impact on MELD prevalence. Fairly in line with other studies [6], MELDs were significantly less frequent among divorced patients. In Switzerland, this group accumulated more hospital days in their last year of life than other unmarried people, even when adjusting for the burden of disease and other sociodemographics [37], suggesting suboptimal health care provision. Discussion of MELDs was less frequent for divorced patients, too, as well as for single patients, however not for widowed patients. This is remarkable, since there is evidence that, irrespective of civil status, up to 95% of people had someone they would trust to make medical decisions for them [38]. However, strong social support and formal proxy decision-makers may be rarer among single and divorced patients, leading to fewer opportunities for communication with physicians.

Place of death had a substantial impact on patient involvement, with shared-decision making being more frequent in hospitals than in nursing homes or at home. This differs from the findings of an international study [39], which found that discussion with patients in most countries was more frequent at home than in institutions.

Unsurprisingly, MELD category had a substantial impact on the prevalence of shared decision-making, with more than 80% involvement of patient/relatives when forgoing and APS are combined. In contrast, in the majority of cases of APS alone, physicians included neither patient nor relatives in the decision-making process, although they perceived their decision as a potentially life-shortening act.

### Limitations

This study has several limitations. Most importantly, the observation unit were deaths and not physicians, i.e., several physicians filled in more than one questionnaire. Due to the anonymous nature of the survey, questionnaires stemming from the same physician could not be identified. Results therefore are not necessarily representative of Swiss physicians. Also, we cannot exclude the possibility of selection bias, since the response rate was, while considerable with respect to the setting, far from 100%. Non-response bias in shared decision-making is also an issue, since 243 questionnaires lacked all information regarding discussion as well as other expression of patient's preferences. However, non-response analysis revealed that the physician's characteristics of this group did not substantially differ from the study population, except an even higher proportion of physicians graduated abroad in the non-response group. The optimal phrasing of the questionnaire remains controversial [40]. This includes the way in which continuous deep sedation is queried, separately from actual MELDs and with less supporting information [41]. However, priority was given to maintain comparability with the international EURELD study [5] and the regular surveys in the Netherlands and Belgium. Finally, the questionnaire does not allow to test whether the actual MELD was in agreement to the preferences of patient and relatives.

## Conclusions

The generally high prevalence of MELDs and shared decision-making in Switzerland support the notion that important goals like doctors' timely anticipation of end-of-life and departure from paternalistic medicine are largely accomplished. While there were few differences between patient groups in terms of MELDs or shared decision-making, divorced patients may be disadvantaged in the decision-making process and subsequent MELDs. Physicians should be proactive about engaging single and divorced patients in shared decision-making, possibly by identifying a proxy well in advance. The association between several physician's attributes and MELD practice points to the possibility of inequity in care and a substantial potential for improvement. The findings that older physicians and those graduated from abroad did not only make fewer MELDs but also if they made an MELD, they discussed it less often with patients, strongly call for additional efforts in residency training programs and physicians' vocational education in order to improve communication skills [42], preferably tailored to address local needs and context [43]. Communication has been called 'the cornerstone of good end-of-life care' [44]. An emphasis should also be given to strengthening physicians' motivation [16] and increasing awareness among patients and relatives that death is near [45], both being important elements in the process of improving end-of-life care.

## Supporting information

S1 Data Patient and physician related determinants for medical end-of-life decisions (N = 3,391). (DTA)

S2 Data Involvement of patient and/or relative(s) in medical end-of-life decisions (N = 2,542). (DTA)
